# Inhibition of Endothelin system during the postnatal nephrogenic period in the rat. Its relationship with hypertension and renal disease in adulthood

**DOI:** 10.1371/journal.pone.0229756

**Published:** 2020-03-03

**Authors:** María Florencia Albertoni Borghese, María del Carmen Ortiz, Rocío C. Marinoni, Lucas H. Oronel, Milena Palamidessi, Carolina A. Yarza, Nicolás Di Siervi, Carlos Davio, Mónica P. Majowicz

**Affiliations:** 1 Departamento de Ciencias Biológicas, Cátedra de Biología Celular y Molecular, Facultad de Farmacia y Bioquímica, Universidad de Buenos Aires, Buenos Aires, Argentina; 2 Departamento de Ciencias Biológicas, Cátedra de Biología Celular y Molecular, CONICET, Facultad de Farmacia y Bioquímica, Universidad de Buenos Aires, Buenos Aires, Argentina; 3 CONICET, Facultad de Farmacia y Bioquímica, Instituto de Investigaciones Farmacológicas (ININFA), Universidad de Buenos Aires, Buenos Aires, Argentina; 4 Departamento de Farmacología, Facultad de Farmacia y Bioquímica, Universidad de Buenos Aires, Buenos Aires, Argentina; Max Delbruck Centrum fur Molekulare Medizin Berlin Buch, GERMANY

## Abstract

The aim of this work was to study the effect of a high sodium (HS) diet on blood pressure and renal function in male adult rats that have been treated with a dual Endothelin receptor antagonist (ERA) during their early postnatal period (day 1 to 20 of life). Male Sprague-Dawley rats were divided in four groups: C_NS_: control rats with normosodic diet; ERA_NS_: ERA-treated rats with normosodic diet; C_HS_: control rats with high sodium diet; ERA_HS_: ERA-treated rats with HS diet. Systolic blood pressure (SBP) was recorded before and after the diet and 24-hour metabolic cage studies were performed. AQP2 and α-ENac expressions were measured by western blot and real time PCR in the renal medulla. Vasopressin (AVP) pathway was evaluated by measuring V2 receptor and adenylyl cyclase 6 (AC6) expression and cAMP production in the renal medulla. Pre-pro ET-1mRNA was also evaluated in the renal medulla. Only rats that had been treated with an ERA during their postnatal period increased their SBP after consumption of a HS diet, showing an impaired capacity to excrete sodium and water, i.e. developing salt sensitivity. This salt sensitivity would be mediated by an increase in renomedullary expression and activity of AQP2 and α-ENaC as a consequence of increased AC6 expression and cAMP production and/or a decreased ET-1 production in the renal medulla. The knowledge of the molecular mechanisms underlying the perinatal programming of salt sensitive hypertension will allow the development of reprogramming strategies in order to avoid this pathology.

## Introduction

The developing embryo and/or fetus is highly sensitive to perturbations of the maternal environment. Adverse environmental factors (nutritional factors, physiologic or psychological stress, endocrine imbalance, ingestion or exposition to drugs among others) can disturb the processes of cell proliferation and differentiation, leading to changes in the normal developmental pathways of mature organs and tissues **[1–3]**. The developing organs can mount an adaptive response in order to ensure survival and the maintenance of critical functions of the tissues. However, these adaptive responses may represent an increased risk for diseases later in life; a process known as “Disease programming” **[3].** The double hit hypothesis proposes that a genetic or environmental first hit during critical periods of development makes an individual more susceptible to a second hit later in life **[4].**

Bearing in mind that renal development in rodents continues along the early postnatal period, not only the fetus is at a risk for developmental disease programming, but also the neonate. Endothelin (ET) has a relevant role during embryonic development since KO animals for any component of ET system have a lethal phenotype **[5–8]**. However, the role of ET system during the postnatal period is not completely understood. We have shown previously that the inhibition of ET system in the rat with a dual ET receptor antagonist (ERA) during the early postnatal period affects both renal structure and function, decreasing the number of glomeruli, the juxtamedullary filtration surface area and the glomerular filtration rate and increasing the proteinuria, being these effects more pronounced in male rats **[9].** It is widely accepted that a reduced glomerular number predisposes to hypertension and to kidney disease in the adulthood **[10–12]**. Brenner and colleagues postulated that reduced filtration surface area associated with a low nephron number would lead to sodium retention and development of systemic hypertension as a compensatory response to maintain sodium homeostasis **[10, 13, 14]**.

The final control of sodium and water reabsorption takes place in the collecting duct (CD) through the amiloride-sensitive epithelial sodium channel (ENaC) and aquaporin-2 (AQP2) water channel respectively **[15]**. Both transporters are regulated by vasopressin (AVP) through V2 receptors **[16]**. Considering that ENaC is responsible for the fine-tuning of sodium reabsorption in the last nephron segment, the role of this channel in sodium reabsorption in the kidney is critical to maintain sodium and volume homeostasis and to control arterial blood pressure **[17–20]**. Excessive AVP-dependent ENaC stimulation could be a risk factor for sodium retention, leading to an increase in blood pressure **[21]**.

ENaC expression and activity is tightly regulated by both aldosterone- dependent and aldosterone-independent mechanisms **[22, 23]**; endocrine as well as local autocrine and paracrine factors play a critical role in the modulation of ENaC, such as ET and purinergic system **[17, 24].** Mice with CD-specific knockout for ET-1 are hypertensive and had reduced sodium excretion in response to sodium loading **[25]**. ET-1 inhibits AVP action at both cortical and medullary CD level, being this effect mediated, at least in part, by PKC-sensitive inhibition of adenylyl cyclase (AC) activity **[26–28]**. Moreover, CD-specific knockout of ET-1 resulted in increased sensitivity to the hydroosmotic and cAMP-stimulating effects of AVP **[29]** and is associated with an increase in AC6 protein abundance **[27],** the protein that mediates AVP-stimulated ENaC activity in the kidney **[30]**.

On these bases, the aim of this work was to study the effect of a high sodium diet in adult rats that have been treated with a dual ERA during their early postnatal period (day 1 to 20 of life). Our hypothesis was that the renal alterations produced by ET system inhibition during the postnatal period predispose to hypertension during adulthood, especially after a second adverse impact, in this case a high sodium diet. This salt sensitivity would be mediated by an increased expression and/or activity of AQP2 and α-ENaC in the animals treated with an ERA during their early postnatal life as a consequence of an exacerbated AVP pathway and/or a decreased renal medullary ET production.

## Materials and methods

### Animals and treatments

Sprague Dawley (SD) rats were purchased from the School of Pharmacy and Biochemistry from the University of Buenos Aires. Protocols were designed according to the National Institutes of Health Guide for the Care and Use of Laboratory Animals, the American Physiological Society “Guiding Principles in the Care and Use of Animals” and to the 6344/96 regulation of Argentinean National Drug Food and Medical Technology Administration (ANMAT) and were approved by the Institutional Committee for Use and Care of Laboratory Animals from the School of Pharmacy and Biochemistry (Cudap N°78096/18; Res D 1388). All rats were housed in rooms with controlled temperature (24°C) and 12 h. dark-light cycle. Food and water were supplied ad libitum. Adult female SD rats (approx. 250 g body weight) were mated by exposure to a fertile SD male during 1 week. After birth, litter size was fixed in 10±1. Litters with less than 9 pups were excluded. Newborn rats were treated daily from postnatal day 1 to postnatal day 20 with vehicle (distilled water) or with Bosentan (Actelion, 20 mg/kg/day), a dual ERA, which was administered orally with a micropipette. Blockade of ET receptors was performed during the first 20 days of life, comprising all the lactation period, since in rats growth and maturation of the kidney also continue after the completion of nephrogenesis and it has been considered that nephrons reach terminal differentiation at the time of weaning **[9].**

After weaning, the animals were allowed to grow up to 65–70 days old and at that point they received a normal sodium diet (NS; 0,3% ClNa) or a high sodium diet (HS; 8% ClNa) **[31–33]** for 8 days. In this study we will only show results corresponding to male groups, so, 4 groups were conformed: C_NS_: control rats with normosodic diet; ERA_NS_: ERA-treated rats with normosodic diet; C_HS_: control rats with high sodium diet; ERA_HS_: ERA-treated rats with high sodium diet.

Food and water consumption was measured daily. Arterial blood pressure was determined before and after the administration of the diets. At the end of the experiment the animals were anesthetized with ketamine/xylacine (100 and 10 mg/kg respectively), blood samples were obtained by cardiac puncture, and the kidneys were immediately excised, weighed and processed.

### Determinations in the 24-hour metabolic cage studies

Twenty four-hour urine samples were collected using metabolic cages. Animals were allowed to acclimatize to metabolic cages for two days and then fasted for 24 h before the collection of urine. Urine samples were analyzed for total protein using a kit provided by Wiener (Proti U/LCR; Wiener Lab., Rosario, Argentina). Urinary and plasmatic sodium and potassium concentrations were evaluated using an ion analyzer (Tecnolab; Mod T-412). Kinetic determinations of serum and urinary creatinine concentrations were evaluated using a kit provided by Wiener (Wiener Lab., Rosario, Argentina). Urine volume was measured gravimetrically.

### Determination of systolic blood pressure

Systolic BP was recorded by triplicate before and after the administration of the normosodic or high sodium diet in conscious rats by tail plethysmography (ADInstruments PowerLab 8/30 and NIBP Controller ML125).

### Histological and histochemical evaluation

The left kidneys were decapsulated and cut longitudinally, fixed in phosphate buffered 10% formaldehyde, pH 7.4, embedded in paraffin wax and cut to a thickness of 5μm. Renal tissue sections were stained with hematoxylin and eosin (H-E) for histological evaluation.

Kidney sections were subjected to Masson's trichrome and Sirius red staining to determine the presence of early fibrosis. At least ten fields of each renal zone from three animals of each group were analyzed.

#### Histochemistry

*Masson´s trichrome staining*. Masson’s trichrome staining was carried out and the proportion of blue-stained fibrotic area in the different renal zones of each section was graded semiquantitatively (0: ≤5%, 1: 5% to 25%, 2: 25% to 50%, 3: 50% to 75%, 4: ≥75%). These examinations were performed blindly by two researchers and the mean values were calculated **[34].**

*Sirius red staining*. Collagen accumulation was examined in the renal sections with the collagen-specific stain picrosirius red (Sirius Red 3 in a saturated aqueous solution of picric acid and fast green as a counterstain). Sirius Red staining is a method for collagen determination, enabling quantitative morphometric measurements to be performed in locally defined tissue areas **[35]**. Staining was scored as 0 (normal and slight staining surrounding the tubular, glomerular, and vascular structures), 1 (weak staining that doubles the normal label surrounding the tubular, glomerular, and vascular structures), 2 (moderate staining in the peritubular interstitium and inside the glomeruli), 3 (strong staining that replaces the glomerular and tubular structures, compromising <25% of the cortical area), or 4 (strong staining that replaces the glomerular and tubular structures, compromising>25% of the cortical area).

*Image capture and analysis*. Images from histological and histochemical sections were captured using a Nikon Alphaphot-2 YS2 light microscope (Nikon Instrument Group, Melville, NY), coupled to a Sony color video camera digital (Model N° SSC-DC50A). All determinations were performed blindly and under similar light, gain and offset conditions by the same researcher.

*Tissue processing for Western blot analysis*. Immediately after the animals were sacrificed, their kidneys were isolated and the renal medulla was dissected and homogenized at 3.000 rpm in an appropriate buffer (250 mmol/l sucrose, 1 mmol/l EDTA, 0.1 mmol/l PMSF and 10 mmol/l Tris-ClH), pH 7.6. Large tissue debris and nuclear fragments were removed by a low-speed spin (1000 g, 10 min, 4°C). Protein concentration was measured using BCA^TM^ Protein Assay Kit (Pierce, Rockford, IL, USA). Absorbances for protein concentration measurements were read using a RT-2100C microplate reader (Rayto, China) at 560 nm.

*Western blots for AQP2 and α-ENaC*. Western blot analysis was used to identify AQP2 and α-ENaC. We evaluated α-ENaC because this is the rate-limiting subunit to form the functional channel **[23].** Blots were incubated overnight at 4°C with the AQP2 antibody (mouse monoclonal anti-rat IgG1 AQP2 [sc 515770]; Santa Cruz Biotechnology, Inc., CA, USA) diluted in blocking solution (1:200), or with α-ENaC antibody (rabbit anti-rat; diluted 1:500; Santa Cruz Biotechnology, Inc. California, USA). Beta-tubulin was used as loading control (rabbit anti-rat beta-tubulin; Abcam Inc., Cambridge, MA, USA). The membranes were then incubated with a donkey anti-rabbit IgG horseradish peroxidase conjugated secondary antibody (1:3000) (Abcam Inc., Cambridge, MA) for α-ENaC and tubulin and were incubated with mIgGκ BP-HRP (sc-516102) HRP conjugated for AQP2 blots (1:2000); Santa Cruz Biotechnology, Inc. California, USA. Blots were visualized using Super SignalTM West Pico Plus chemiluminescent substrate (Thermo Scientific; Rockford, IL, USA).

The relative protein levels were determined by analyzing the bands with Gel Pro Analyzer 3.1 for Windows and relative protein expression was calculated as the ratio protein of interest/β-tubulin. The AQP2 antibody recognizes a 28-kDa band corresponding to unglycosylated AQP2 and bands between 35–40 kDa representing glycosylated forms of the protein. The α-ENaC antibody recognizes a 78-kDa band and the β-tubulin antibody recognizes a 50kDa band.

*Real-time PCR for AQP2*, *α-ENaC*, *V*_*2*_ *receptor*, *adenylyl cyclase-6 and Pre-pro-ET-1*. Total RNA was isolated using the SV total RNA Isolation System (Promega, Madison, WI, USA). Total RNA was reverse transcribed to cDNA using a high capacity reverse transcription kit (A&B, CA, USA). For real-time detection of AQP2 transcripts and the reference gene (GAPDH), MezclaReal (Real Time PCR commercial mixture from Biodynamics, Argentina) and specific primers were used **[36, 37]**.

The normalized gene expression method (2^–ΔΔCT^) for the relative quantification of gene expression was used. The difference in the cycle threshold (CT) for AQP2 and GAPDH for the control untreated rats was substracted from the difference in the CT for AQP2 and GAPDH for each of the experimental groups **[38]**. The following formula was applied:

ΔΔCT=(CTAQP2−CTGAPDH)experimental−(CT AQP2−CT GAPDH)control untreated rats).

The real-time PCR started at 94°C for 2 min and was followed by 35 thermal cycles at 94°C for 15 s, 58°C for 35 s and 72°C for 30 s.

*cAMP measurements*. cAMP was measured in the renal medulla. Approximately 100 mg of renal medulla was homogenized in ice-cold absolute ethanol and centrifuged for 15 min at 1200g. The supernatant was dried, and the remaining residue was suspended for cAMP determination by competition of [3H]-cAMP for PKA **[39]**. Results were expressed as pmoles cAMP/mg of protein.

*Statistics*. Two-way ANOVA with Bonferroni´s post-test for multiple comparisons was performed using Graph Pad Prism version 5.0 for Windows.

## Results

### Characterization of the experimental model

**Fig 1:**
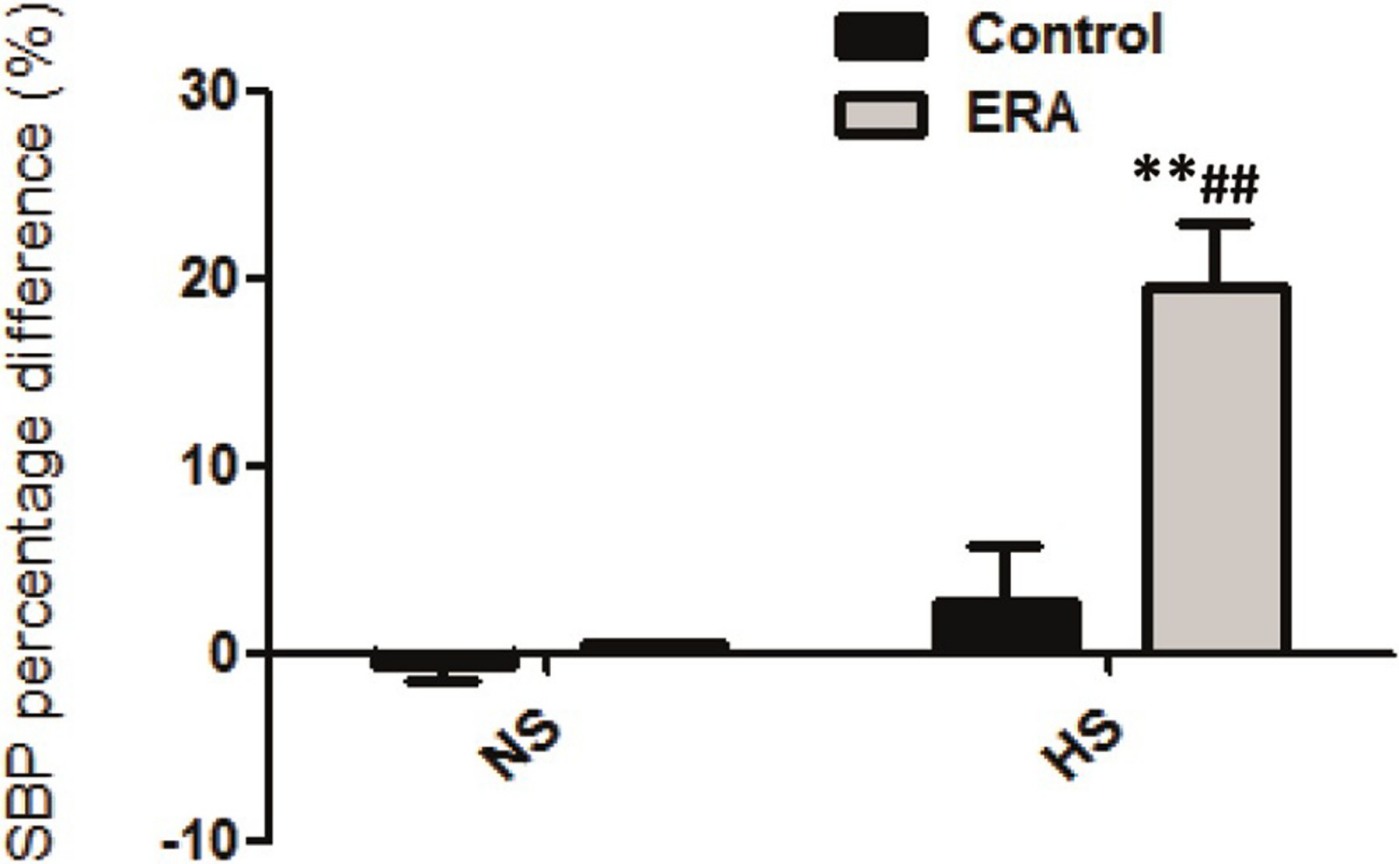
Systolic blood pressure percentage difference (%). The change in systolic blood pressure after a normal or high sodium diet in control and in ERA-treated rats is shown as the percentage difference. ERA: Endothelin receptor antagonist; NS: normosodic diet; HS: high sodium diet (8% NaCl). **p<0.01 vs C_HS_; ##p<0.01 vs ERA _NS._ Data are mean ± SEM of eight independent determinations.

As can be seen in Fig 1, systolic blood pressure (SBP), expressed as SBP percentage difference, only increased significantly (20% increment) in ERA_HS_ vs both C_HS_ and ERA_NS_ (p<0.01).

Table 1 shows weights and consumptions for the different experimental groups. There were no significant changes in body weight (b.w.) or femur length among experimental groups. However, there was a significant increase in renal weight expressed as g/100g of body weight in both C_HS_ and ERA_HS_ when compared with their respective NS controls (p<0.01 in both cases). Food intake, expressed per 100g b.w. was similar in all groups of rats, suggesting that the differences seen in blood pressure were not due to different sodium intakes. As expected, water intake significantly increased in the groups that received HS diet, being this increment of the same magnitude in both groups (p<0.001 vs NS groups).

**Table 1:** Weights and consumptions.

	C_NS_	ERA_NS_	C_HS_	ERA_HS_
Body weight (g)	376±14	380±11	371±13	370±9
Femur lenght (cm)	3.38±0.03	3.35±0.07	3.36±0.02	3.40±0.03
Renal weight (g/100g b.w.)	0.73±0.02	0.72±0.01	0.78±0.01**	0.76±0.02##
Food intake (g/100g b.w.)	6.9±0.3	6.7±0.1	6.5±0.1	6.7±0.2
Water intake (ml/100g b.w.)	7.4±0.3	8.4±0.5	22.6±0.6***	23.2±1.0###

ERA: Endothelin receptor antagonist. C_NS_: control rats with normosodic diet; ERA_NS_: ERA-treated rats with normosodic diet; C_HS_: control rats with high sodium diet; ERA_HS_: ERA-treated rats with high sodium diet. Data were analyzed using two-way ANOVA followed by Bonferroni posttest.

**p<0.01 vs C_NS_;

***p<0.001 vs C_NS_;

##p<0.01 vs ERA_NS_;

###p<0.001 vs ERA_NS._ Data are mean ± SEM of 10 independent determinations, except for femur length, with 6 independent determinations.

### Renal and plasmatic functional parameters

Diuresis significantly increased in both groups that received HS diet when compared with their respective NS groups (*p<0.05 C_HS_ vs C_NS_; #p<0.05 ERA_HS_ vs ERA _NS_). However, this increment was of lower magnitude in ERA_HS_ than in C_HS_ rats. Fractional sodium excretion significantly increased in both groups that received HS diet when compared with their respective NS groups (***p<0.001 vs C_NS_; ###p<0.001 vs ERA _NS_). However, this increment was significantly lower (&p<0.05 vs C_HS_) in ERA_HS_ than in C_HS_ rats. Kaliuresis was significantly lower in both HS groups when compared with their respective NS controls (**p<0.01 C_HS_ vs C_NS_; ## p<0.01 ERA_HS_ vs ERA_NS_). Creatinine clearance significantly increased in both HS groups when compared with their respective NS controls (*p<0.05 C_HS_ vs C_NS_; # p<0.05 ERA_HS_ vs ERA_NS_). Proteinuria significantly increased in both groups that received HS diet vs their respective controls with NS diet (p<0.05 in both cases). Note that the values of proteinuria were higher (although not significant; p = 0.1182) in the rats treated postnatally with the ERA when compared with both groups of control rats. Natremia increased in ERA_HS_ when compared with C_HS_ rats (&p<0.05) while kalemia had a tendency to increase in both HS groups vs their respective NS controls (p = 0.1137). The results correspondents to renal and plasmatic parameters are shown in Table 2.

**Table 2:** Renal and plasma functional parameters.

	C_NS_	ERA_NS_	C_HS_	ERA_HS_
Diuresis (ml/24 h/100g b.w.)	1.9±0.5	2.2±0.4	3.5±0.6*	2.9±0.5#
Fractional Na^+^ excretion (FENa %)	0.43±0.03	0.36±0.03	1.37±0.15***	1.10±0.12###&
Kaliuresis (meq/24 h/100g b.w)	0.38±0.02	0.51±0.13	0.24±0.07**	0.23±0.06##
Creatinine clearance (ml/min/100g b.w.)	0.28±0.04	0.37±0.04	0.42±0.05*	0.53±0.06#
Proteinuria (mg/24 h/100g b.w.)	2.8±0.5	3.6±0.5	4.0±0.5*	4.9±0.5#
Natremia (meq/L)	143±1	145±1	141±2	148±2&
Kalemia (meq/L)	4.2±0.3	4.3±0.4	4.6±0.8	5.2±0.3

ERA: Endothelin receptor antagonist. C_NS_: control rats with normosodic diet; ERA_NS_: ERA-treated rats with normosodic diet; C_HS_: control rats with high sodium diet; ERA_HS_: ERA-treated rats with high sodium diet. Data were analyzed using two-way ANOVA followed by Bonferroni posttest.

*p<0.05 vs C_NS_;

**p<0.01 vs C_NS_

***p<0.001 vs C_NS_;

#p<0.05 vs ERA_NS_;

##p<0.01 vs ERA_NS_

###p<0.001 vs ERA_NS_;

& p<0.05 vs C_HS._ Data are mean ± SEM of 9 independent determinations.

### Histological and histochemical evaluation

The histological structure of rat kidneys in the H-E sections seemed to be unaffected (S1 Fig). The score for both Masson´s trichrome and Sirius red staining was <1 for all the groups in the different renal zones (Table 3); it means a normal and slight staining surrounding tubular, glomerular and vascular structures. There was a significant effect of the HS diet on Masson´s trichrome staining in the renal cortical and juxtamedullary areas. However, the scores were < 1. Representative images are shown in S2, S3, S4, S5 and S6 Figs.

**Table 3:** Histochemical evaluation: Masson’s trichrome and Sirius red staining.

	C_NS_	C_HS_	ERA_NS_	ERA_HS_
Masson´s trichrome score (CA)	0.70±0.11	0.95±0.05*	0.80±0.09	0.90±0.07#
Masson´s trichrome score (JA)	0.75±0.10	0.96±0.05*	0.81±0.09	0.91±0.07#
Masson´s trichrome score (Medulla)	0.65±0.11	0.70±0.11	0.75±0.10	0.80±0.09
Masson´s trichrome score (Papilla)	0.33±0.13	0.41±0.12	0.46±0.14	0.67±0.14
Sirius red score (CA)	0.35±0.11	0.50±0.11	0.45±0.11	0.43±0.11
Sirius red score (JA)	0.80±0.09	0.90±0.10	0.85±0.08	0.90±0.07
Sirius red score (Medulla)	0.55±0.11	0.59±0.11	0.55±0.11	0.65±0.11
Sirius red score (Papilla)	0.33±0.13	0.44±0.13	0.30±0.15	0.71±0.13

ERA: Endothelin receptor antagonist. C_NS_: control rats with normosodic diet; ERA_NS_: ERA-treated rats with normosodic diet; C_HS_: control rats with high sodium diet; ERA_HS_: ERA-treated rats with high sodium diet. Data were analyzed using two-way ANOVA followed by Bonferroni posttest.

*p<0.05 vs C_NS_;

#p<0.05 vs ERA_NS._ Data are expressed as mean ± SEM. n = 3 rats/group and at least 10 fields/animal were analyzed.

### AQP2 and α-ENaC expression

**Fig 2:**
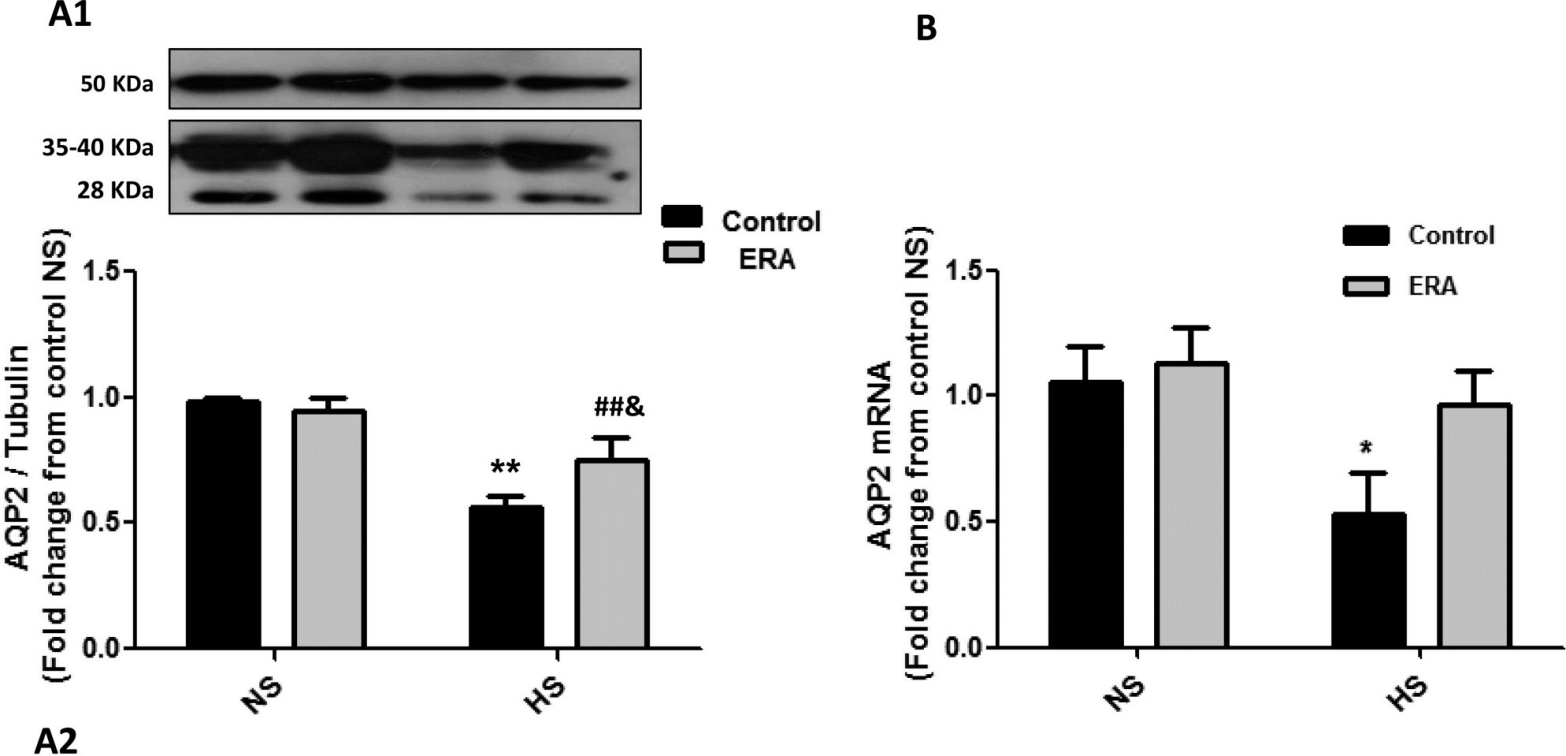
AQP2 expression in the renal medulla. **A1.** Representative Western Blot analysis of AQP2 (28 and 35–40 kDa) and tubulin (50 kDa) in homogenates of the renal medulla. **A2.** AQP2 protein expression indicated as AQP2/tubulin ratio fold change from C_NS_ rats. **B.** AQP2 mRNA levels are expressed as relative values from C_NS_ rats. The following formula was applied: ΔΔCT = (CTAQP2-CTGAPDH) experimental–(CTAQP2-CT GAPDH) C_NS_ rats. ERA: Endothelin receptor antagonist; NS: normosodic diet; HS: high sodium diet (8% NaCl). Data were analyzed using Two-way ANOVA followed by Bonferroni posttest. **p<0.01 vs C_NS_; ##p<0.01 vs ERA_NS_; &p<0.05 vs C_HS_._._ Data are mean ± SEM of four independent determinations.

**Fig 3:**
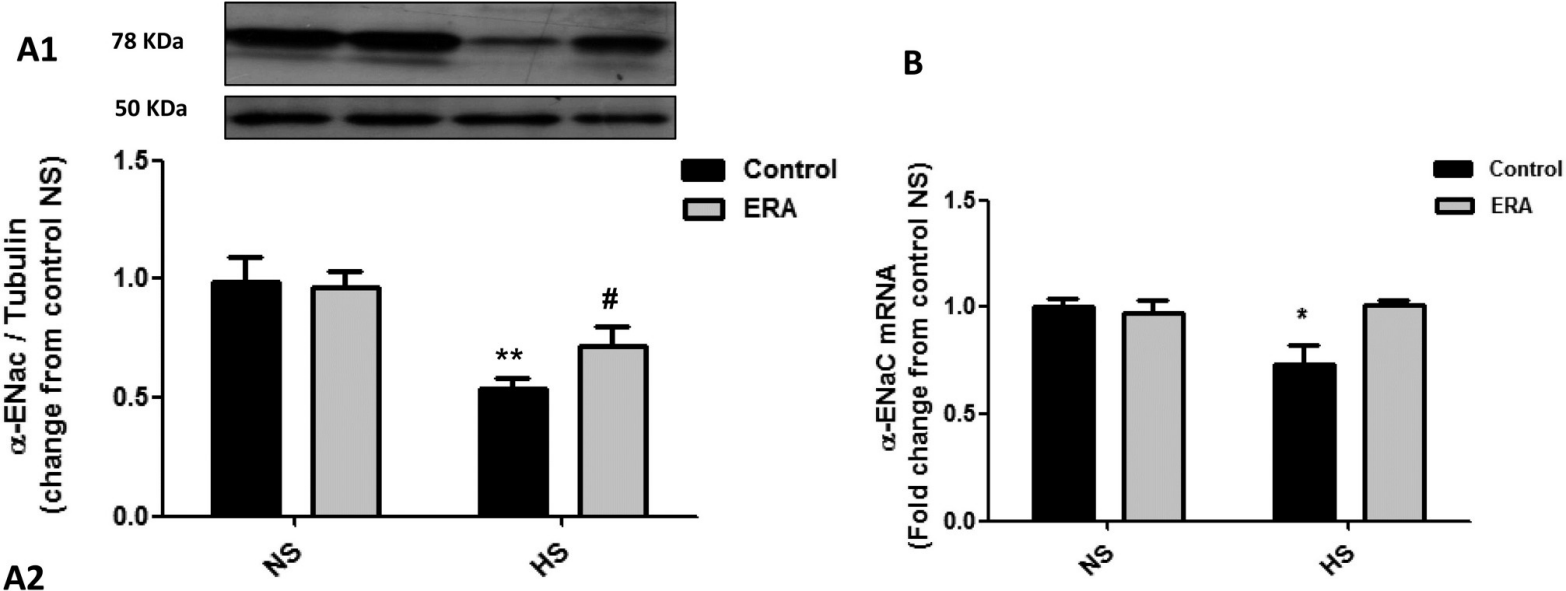
α-ENaC expression in the renal medulla. **A1.** Representative Western Blot analysis of α-ENaC (78 kDa) and tubulin (50 kDa) in homogenates of the renal medulla. **A2.** α-ENaC protein expression indicated as α-ENaC /tubulin ratio fold change from C_NS_ rats. **B.** α-ENaC mRNA levels are expressed as relative values from C_NS_ rats. The following formula was applied: ΔΔCT = (CT α-ENaC -CTGAPDH) experimental–(CT α-ENaC -CT GAPDH) C_NS_ rats. ERA: Endothelin receptor antagonist; NS: normosodic diet; HS: high sodium diet (8% NaCl). Data were analyzed using Two-way ANOVA followed by Bonferroni posttest. *p<0.05 vs C_NS_; #p<0.05 vs C_HS_._._ Data are mean ± SEM of four independent determinations.

AQP2 mRNA expression significantly decreased in C_HS_ group when compared with C_NS_ (p< 0.05), meanwhile failed to decrease in ERA_HS_. Moreover, AQP2 protein expression significantly decreased in C_HS_ group when compared with C_NS_ (p< 0.01) and in ERA_HS_ group when compared with ERA_NS_ (p<0.01). However, the expression of AQP2 protein was significantly higher in ERA_HS_ when compared with C_HS_ (p<0.05). These results can be seen in Fig 2A and 2B. α-ENaC mRNA expression significantly decreased in C_HS_ group when compared with C_NS_ (p< 0.05), meanwhile failed to decrease in ERA_HS_. Moreover, α-ENaC protein expression significantly decreased in C_HS_ group when compared with C_NS_ (p< 0.05) and in ERA_HS_ group when compared with ERA_NS_ (p<0.05). However, the expression of α-ENaC protein was higher although not significant (p = 0.1138) in ERA_HS_ (0.72±0.05) when compared with C_HS_ (0.54±0.03). In fact, the administration of HS diet decreased 45.5% α-ENaC protein expression in control rats while decreased 25% α-ENaC protein expression in ERA rats. These results can be seen in Fig 3A and 3B.

The results related to AQP2 and α-ENaC in combination with those obtained when we evaluated renal parameters suggest that ERA_HS_ rats have an impaired ability to excrete water and sodium.

### Participation of AVP pathway on AQP2 and α-ENaC altered expression in ERA_HS_

Bearing in mind that AVP regulates both AQP2 and α-ENaC **[16]**, we decided to evaluate if this pathway was implicated in our experimental model, so we evaluated V2 receptor mRNA expression, AC6 mRNA expression and renomedullary cAMP production.

### AVP-V2 receptor expression

**Fig 4:**
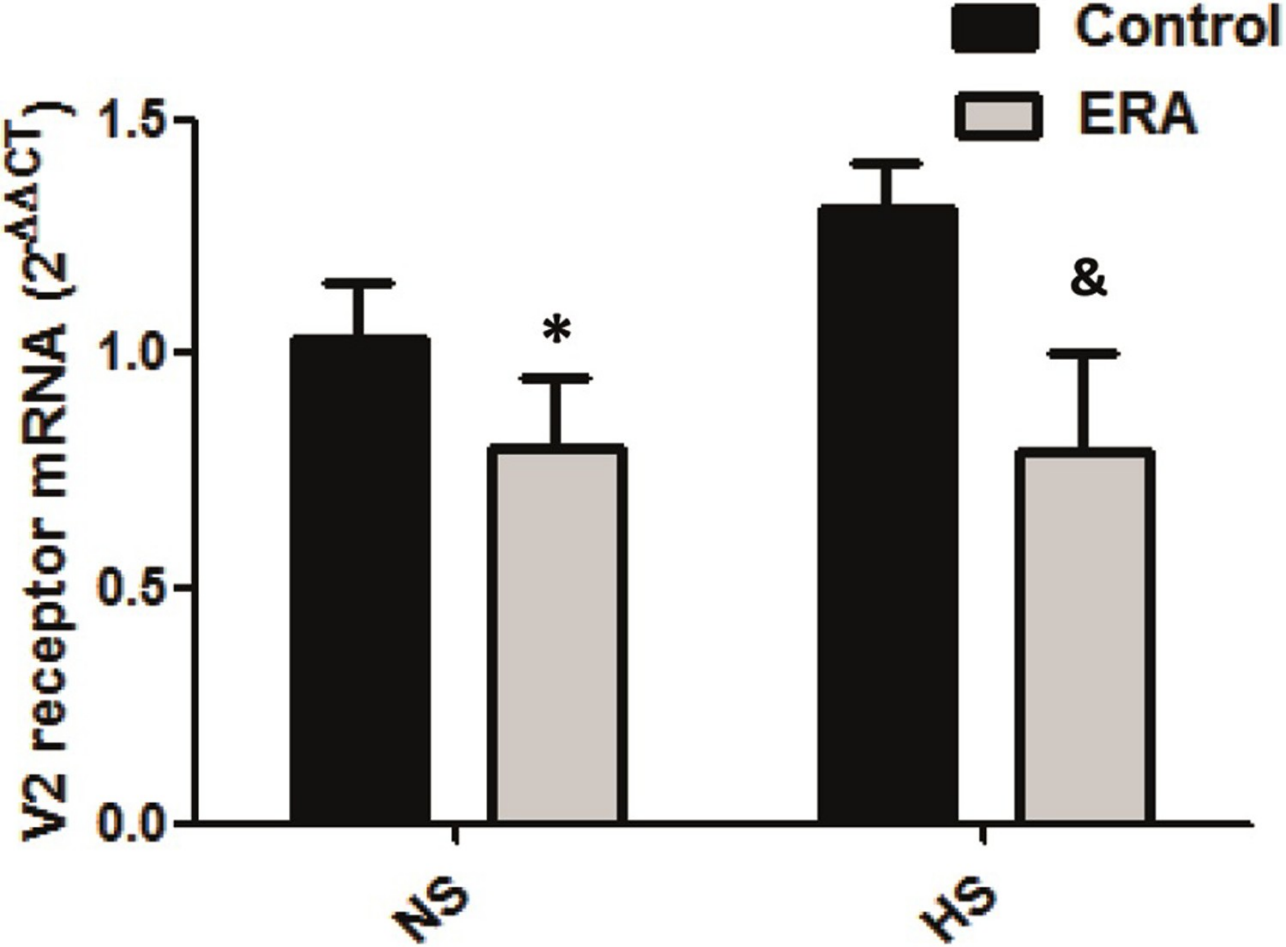
Vasopressin V2 receptor expression in the renal medulla. Vasopressin V2 receptor mRNA levels are expressed as relative values from C_NS_ rats. The following formula was applied: ΔΔCT = (CTV2 receptor-CTGAPDH) experimental–(CTAV2 receptor-CT GAPDH) C_NS_ rats. ERA: Endothelin receptor antagonist; NS: normosodic diet; HS: high sodium diet (8% NaCl). Data were analyzed using Two-way ANOVA followed by Bonferroni posttest. *p<0.05 vs C_NS_; &p<0.05 vs C_HS_._._ Data are mean ± SEM of five independent determinations.

V2 receptor mRNA expression was significantly lower in ERA_NS_ vs C_NS_ (p<0.05) and in ERA_HS_ vs C_HS_ (p<0.05), suggesting that the increased expression of AQP2 and α-ENaC in ERA_HS_ rats would not be due to a greater level of V2 receptor expression. This result can be seen in Fig 4.

### AC6 mRNA expression

**Fig 5:**
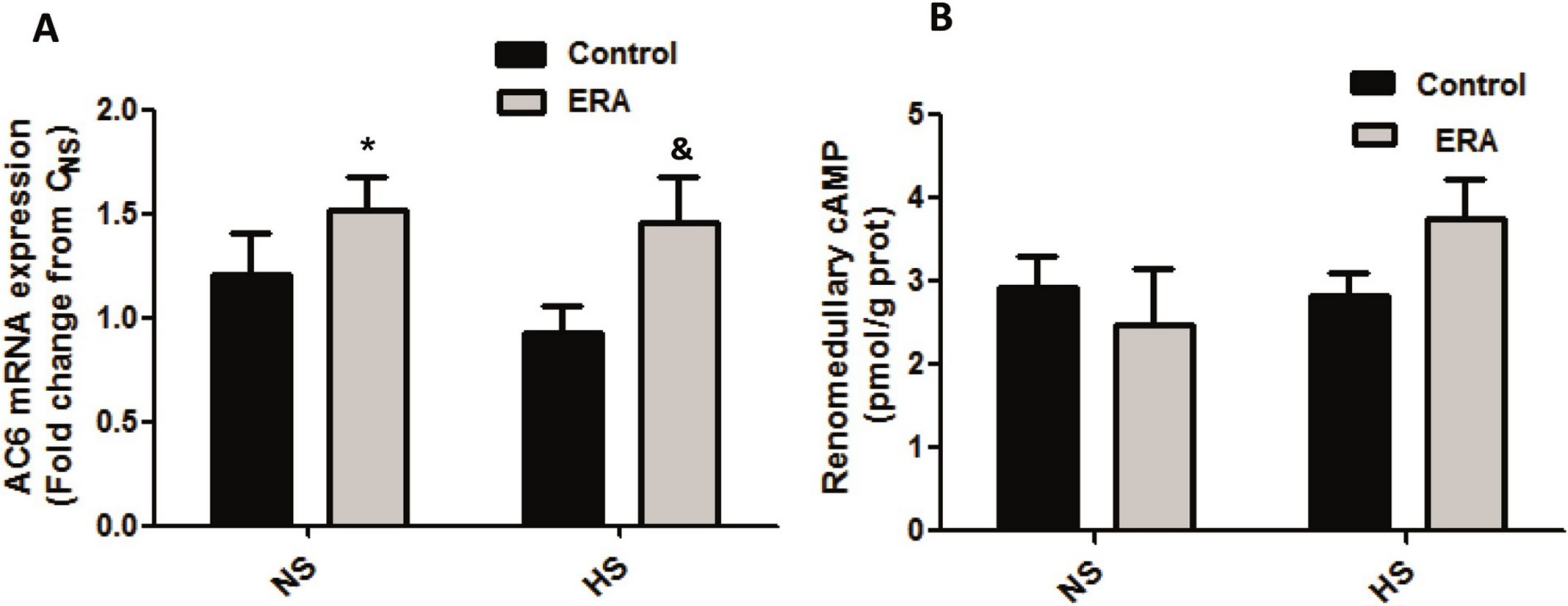
Adenylyl cyclase 6 expression and cAMP production in the renal medulla. **A.** Adenylyl cyclase 6 (AC6) mRNA levels are expressed as relative values from C_NS_ rats. The following formula was applied: ΔΔCT = (CTAC6-CTGAPDH) experimental–(CTAC6 receptor-CT GAPDH) C_NS_ rats. ERA: Endothelin receptor antagonist; NS: normosodic diet; HS: high sodium diet (8% NaCl). Data were analyzed using Two-way ANOVA followed by Bonferroni posttest. *p<0.05 vs C_NS_; &p<0.05 vs C_HS_._._ Data are mean ± SEM of four independent determinations. **B.** cAMP production assessed by competition of [3H]-cAMP for PKA. ERA: Endothelin receptor antagonist; NS: normosodic diet; HS: high sodium diet (8% NaCl). Data were analyzed using Two-way ANOVA followed by Bonferroni posttest. Data are mean ± SEM of five independent determinations.

AC6 mRNA expression increased in ERA_HS_ vs C_HS_ group (p<0.05). Besides, ERA_NS_ group had a higher level of AC6 expression than C_NS_ (p<0.05). This result can be seen in Fig 5A.

### Renomedullary cAMP production

There were no significant differences between groups in renomedullary cAMP production. However, there was a tendency to increase cAMP production (expressed as pmoles cAMP/g protein) in ERA_HS_ when compared with ERA_NS_ (p = 0.1655) rats meanwhile that tendency was not seen in C_HS_ when compared with C_NS_ (p = 0.8204). Although cAMP production in ERA_HS_ was not significantly different from the value obtained for C_HS_, it was 25% higher. Besides, cAMP production in ERA_HS_ was 51% higher than in ERA_NS_ (C_NS_: 2.93±0.36; ERA_NS_: 2.47±0.69; C_HS_: 2.82±0.29; ERA_HS_: 3.74±0.49). These results can be seen in Fig 5B.

Regarding AC6 expression in our experimental model, it was significantly increased in both groups of ERA-treated rats. However, renomedullary cAMP had a tendency to increase only in ERA _HS_ rats, suggesting a greater activity of AC6 in this group. Thus it is probably that HS diet differently regulates AC6 activity in ERA-treated rats than in control rats. We must consider that 8 days is an acute period of time; possibly a chronic salt consumption will show more significant changes.

### Pre-pro ET-1 production is decreased in the renal medulla of ERA_HS_ rats

**Fig 6:**
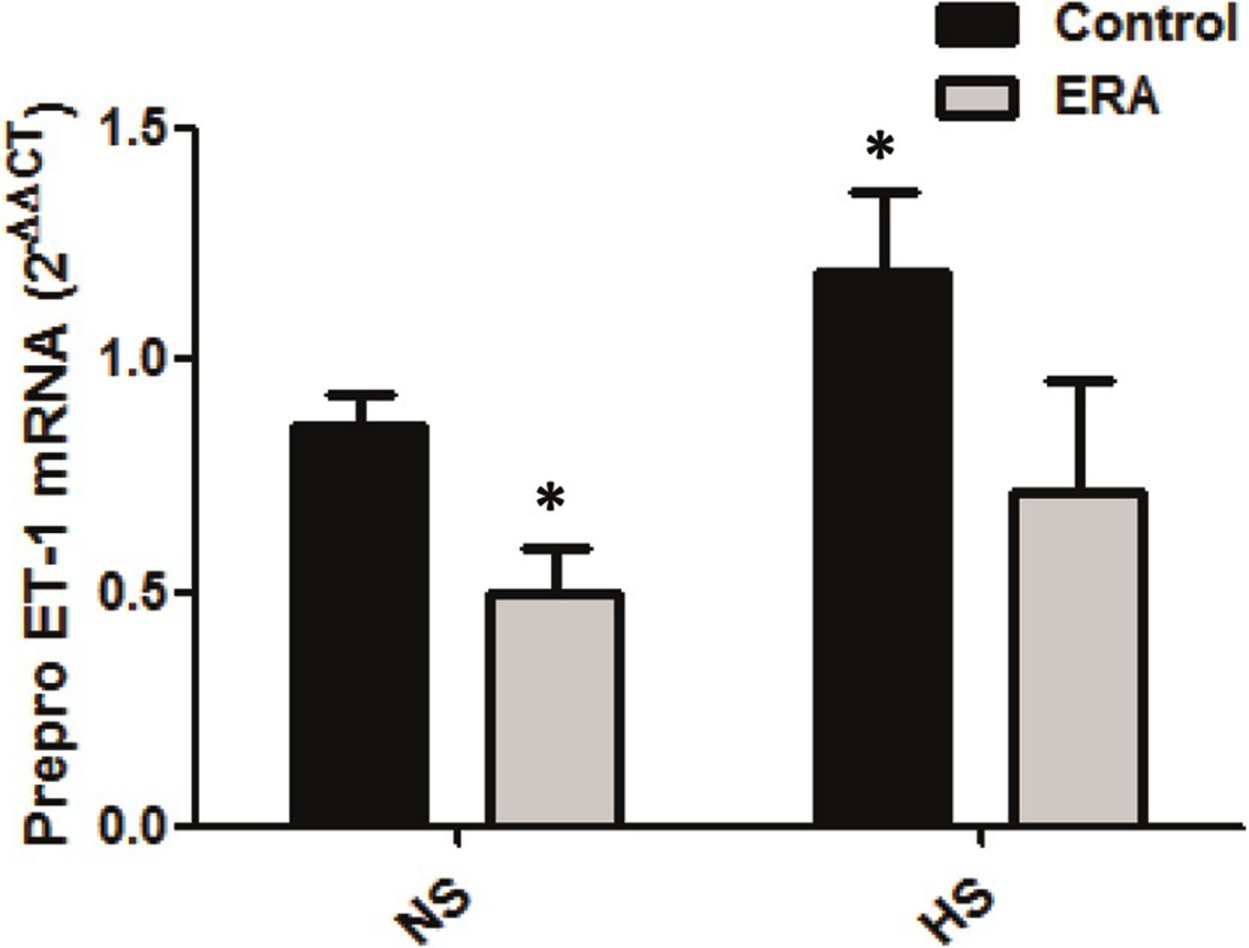
Pre-pro ET-1 expression in the renal medulla. Pre-pro ET-1 receptor mRNA levels are expressed as relative values from C_NS_ rats. The following formula was applied: ΔΔCT = (CT Pre-pro ET-1 -CTGAPDH) experimental–(CT Pre-pro ET-1 receptor-CT GAPDH) C_NS_ rats. ERA: Endothelin receptor antagonist; NS: normosodic diet; HS: high sodium diet (8% NaCl). Data were analyzed using Two-way ANOVA followed by Bonferroni posttest. *p<0.05 vs C_NS_._._ Data are mean ± SEM of four independent determinations.

Bearing in mind that medullary ET-1 is fundamentally important in physiologic regulation of renal sodium and water excretion and maintenance of normal systemic blood pressure, we measured mRNA pre-pro ET-1 by real time PCR. As expected, pre-pro ET-1 significantly increased in C_HS_ vs C_NS_ rats (p<0.05). On the other hand, ERA_NS_ showed significantly lower expression of pre-pro ET-1 when compared with C_NS_ rats (p<0.05) and besides, ERA_HS_ failed to increase pre-pro ET-1 expression when compared with ERA_NS_ rats. These results can be observed in Fig 6.

## Discussion

In this paper we show that adult male SD rats that had been treated with a dual ERA during their early postnatal period have a 20% increase in their blood pressure after 8 days consuming a high sodium diet. We had shown in a previous paper that the inhibition of the ET system with a dual ERA produces a decrease in nephron number **[9].** Although low nephron number is not always associated with hypertension, offspring with diminished nephron number are more susceptible to a second insult or adverse impact **[40].** The increase in blood pressure seen in ERA_HS_ rats was not due to higher food intake because there were no significant differences in this parameter among the different experimental groups. Besides, there was neither significant difference in body weight nor in femur length among the different experimental groups. High salt intake increased proteinuria in both control and ERA-treated rats in a similar magnitude (p<0.05) but ERA-treated rats on a NS diet already had a proteinuria 28% higher (although not significant; p = 0.1182) than control rats on a NS diet. Thus it is possible that the composition and/or the function of the glomerular filtration barrier had been affected during postnatal development in ERA-treated rats. In fact, it has been shown that both glomerular endothelial cells and podocytes express ET receptors and synthetize ET-1 and there is a cross- talk between these two cell types that may be pathologic if there is an imbalance in the ET system **[41, 42]**.

On the other hand, ERA_HS_ rats showed a decreased ability to eliminate sodium and water when compared with C_HS_ group. This decreased ability to excrete sodium and water is in line with the higher plasmatic sodium levels and concomitantly with the higher blood pressure in ERA_HS_ group.

Bearing in mind that the final control of sodium and water reabsorption takes place in the CD through ENaC and AQP2 respectively **[15]**, we evaluated the expression of these transporters. As expected, the expression of both α-ENaC and AQP2 mRNA and proteins decreased in C_HS_ vs C_NS_ rats but failed to decrease or decreased at a lower extent in ERA_HS_ rats. These and the above mentioned results suggest that ERA_HS_ rats have a decreased ability to excrete sodium and water during a sodium overload due to a higher level expression of sodium and water transporters ENaC and AQP2. In fact, ERA_HS_ showed a lower diuresis and fractional sodium excretion than C_HS_ rats. The lower diuresis and fractional sodium excretion in ERA_HS_ was not due to a decreased glomerular filtration rate because creatinine clearance was even higher in both HS groups when compared with their respective controls. Thus the decreased ability to excrete sodium and water during a sodium overload in ERA_HS_ rats would be due to increased sodium and water reabsorption mediated by ENaC and AQP2 respectively at CD level. Another result of the current study is that rats treated with a high sodium diet have a decreased ability to excrete K^+^. This result is in accordance with Jensen et al, who provide experimental data showing that ENaC activity is a rate-limiting element for powerful K^+^ excretion, hindering K^+^ excretion during high Na^+^ conditions **[43]**.

It is well known that AVP, through V2-receptors, is the main regulator of AQP2, stimulating both its expression and translocation and thus promoting water reabsorption at CD level **[44]**. However, in the last years, it has re-emerged the concept that the ability of AVP to stimulate water reabsorption is possible by promoting discretionary sodium reabsorption via ENaC along the distal nephron and consequently decreasing sodium excretion **[16].** Thus AVP uses V2 receptors coupled to G_s_ and stimulation of AC and production of cAMP as a common signaling pathway to increase both ENaC and AQP2 expression and activity **[16].** In the current study we show that V2 receptor mRNA expression was decreased in ERA_HS_ rats, thus the increased α-ENaC expression and decreased ability to excrete sodium was not consequence of an increased V2 receptor expression in our experimental model. However, we found that AC6 expression was increased and renomedullary cAMP had a marked tendency to increase in ERA_HS_ rats when compared with C_HS_, so these results suggest that the increased ENaC and AQP2 expression seen in ERA_HS_ rats would be a consequence of increased AC6 and concomitantly increased cAMP production in the renal medulla. In fact, AC6 in the CD regulates renal water excretion, most likely through control of AVP-stimulated cAMP accumulation and AQP2 **[45]** and it was recently shown that AC6 mediates AVP-stimulated ENaC activity in the kidney **[30].**

Another interesting result of the current study is that renomedullary Pre-pro-ET-1 production was decreased in ERA_HS_ when compared with C_HS_. In fact, as expected, we found an increment in renomedullary Pre-pro-ET-1 production in control rats but we failed to find this increment in ERA rats after the administration of the high sodium diet. It is well-known that medullary ET-1 is fundamentally important in physiologic regulation of renal sodium and water excretion and maintenance of normal systemic blood pressure **[46]**. Mice with CD-specific knockout of the ET-1 gene have impaired sodium excretion in response to sodium loading and have hypertension which worsens with high salt intake **[25]**. Strait et al showed that CD ET-1 KO IMCD had greater sensitivity to forskolin than did control IMCD, suggesting that neither the V2 receptor nor G proteins can account for the increased cAMP levels in CD ET-1 KO mice, supporting a primary change in AC activity per se. They concluded that due to the known acute inhibitory effect of ET-1 on AVP-stimulated cAMP accumulation CD-derived ET-1 might exert a diuretic effect through both acute modulation of AC activity and chronic down-regulation of AC protein content **[27]**. Therefore, the enhanced AVP pathway in the ERA-treated rats that received a HS diet may be a consequence of the decreased renomedullary ET-1 production.

## Conclusions

The results of the present study provide evidence that the inhibition of ET system during the early postnatal period in rodents could predispose to salt sensitive hypertension during adulthood. This salt sensitivity would be mediated by an increased renomedullary expression and activity of AQP2 and α-ENaC as a consequence of increased AC6 expression and cAMP production and/or a decreased ET-1 production in the renal medulla.

The knowledge of the molecular mechanisms underlying the perinatal programming of salt sensitive hypertension will allow the development of reprogramming strategies in order to prevent this pathology.

## Supporting information

S1 Raw images (PDF)

S1 Fig Hematoxylin-eosin staining of the renal cortex. Representative images for Hematoxylin-eosin staining of the renal cortex. **A =** C_NS_ (CA); B = C_HS_ (CA); **C =** ERA_NS_ (CA); **D =** ERA_HS_ (CA); **E =** C_NS_(JA); **F =** C_HS_ (JA); **G =** ERA_NS_ (JA); **H =** ERA_HS_ (JA). C: control; ERA: Endothelin receptor antagonist. NS: normosodic diet; HS: high sodium diet. CA: cortical area; JA: juxtamedullary area. Total magnification: 400x.
(TIF)

S2 Fig Hematoxylin-eosin staining of the renal medulla and papilla. Representative images for Hematoxylin-eosin staining of the renal medulla (A-D) and the renal papilla (E-H). **A =** C_NS_; **B =** C_HS_; **C =** ERA_NS_; **D =** ERA_HS_; **E =** C_NS_; **F =** C_HS_; **G =** ERA_NS_; **H =** ERA_HS_. C: control; ERA: Endothelin receptor antagonist. NS: normosodic diet; HS: high sodium diet. Total magnification: 400x.
(TIF)

S3 Fig Masson's trichrome staining of the renal cortex. Representative images for Masson's trichrome staining of the renal cortex. **A =** C_NS_ (CA); **B =** C_HS_ (CA); **C =** ERA_NS_ (CA); **D =** ERA_HS_ (CA); **E =** C_NS_(JA); **F =** C_HS_ (JA); **G =** ERA_NS_ (JA); **H =** ERA_HS_ (JA) C: control; ERA: Endothelin receptor antagonist. NS: normosodic diet; HS: high sodium diet. CA: cortical area; JA: juxtamedullary area. Total magnification: 400x.
(TIF)

S4 Fig Masson's trichrome staining of the renal medulla and papilla. Representative images for Masson trichrome staining of the renal medulla (A-D) and the renal papilla (E-H). **A =** C_NS_; **B =** C_HS_; **C =** ERA_NS_; **D =** ERA_HS_; **E =** C_NS_; **F =** C_HS_; **G =** ERA_NS_; **H =** ERA_HS_ C: control; ERA: Endothelin receptor antagonist. NS: normosodic diet; HS: high sodium diet. Total magnification: 400x.
(TIF)

S5 Fig Sirius red staining of the renal cortex. Representative images for Sirius red staining of the renal cortex. **A =** C_NS_ (CA); **B =** C_HS_ (CA); **C =** ERA_NS_ (CA); **D =** ERA_HS_ (CA); **E =** C_NS_(JA); **F =** C_HS_ (JA); **G =** ERA_NS_ (JA); **H =** ERA_HS_ (JA) C: control; ERA: Endothelin receptor antagonist. NS: normosodic diet; HS: high sodium diet. CA: cortical area; JA: juxtamedullary area. Total magnification: 400x.
(TIF)

S6 Fig Sirius red staining of the renal medulla and papilla. Representative images for Sirius red staining of the renal medulla (A-D) and the renal papilla (E-H). **A =** C_NS_; **B =** C_HS_; **C =** ERA_NS_; **D =** ERA_HS_; **E =** C_NS_; **F =** C_HS_; **G =** ERA_NS_; **H =** ERA_HS_; C: control; ERA: Endothelin receptor antagonist. NS: normosodic diet; HS: high sodium diet. Total magnification: 400x.
(TIF)
