# Supplementary material for: Inhibition of Endothelin system during the postnatal nephrogenic period in the rat. Its relationship with hypertension and renal disease in adulthood
Source: PLoS One. 2020 Mar 3;15(3):e0229756. doi: 10.1371/journal.pone.0229756 (PMC7053749; doi:10.1371/journal.pone.0229756)

Fig 2: AQP2 protein expression

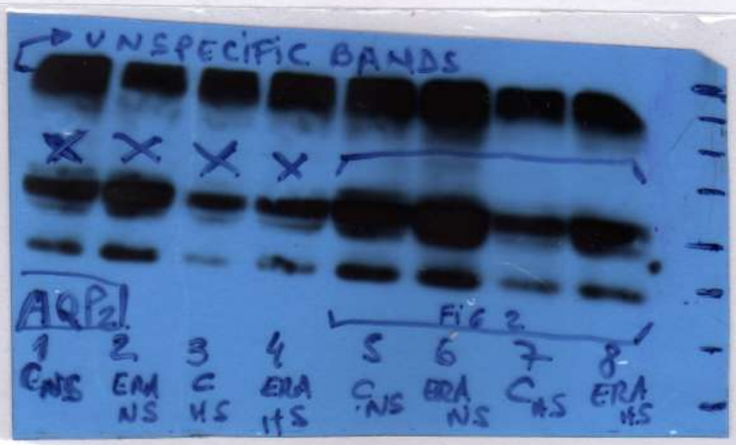

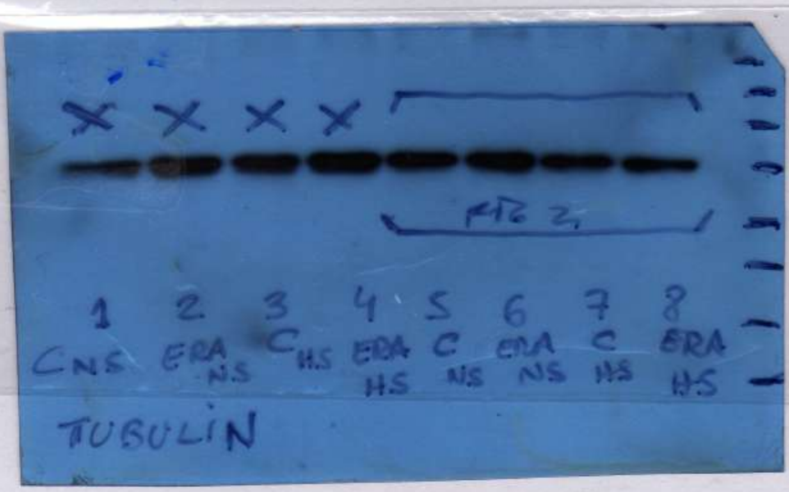

CNS: Control / normosodic diet

CHS: Control / high sodium diet

ERA_{NS}: endothelin receptor antagonist treatment / normosodic diet

ERAHS: endothelin receptor antagonist treatment / high sodium diet

Fig 3- α-ENaC expression

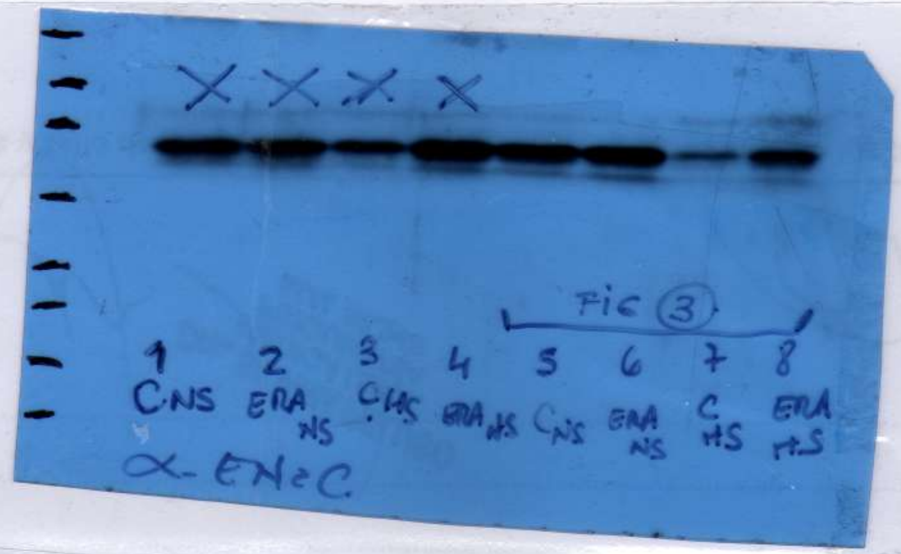

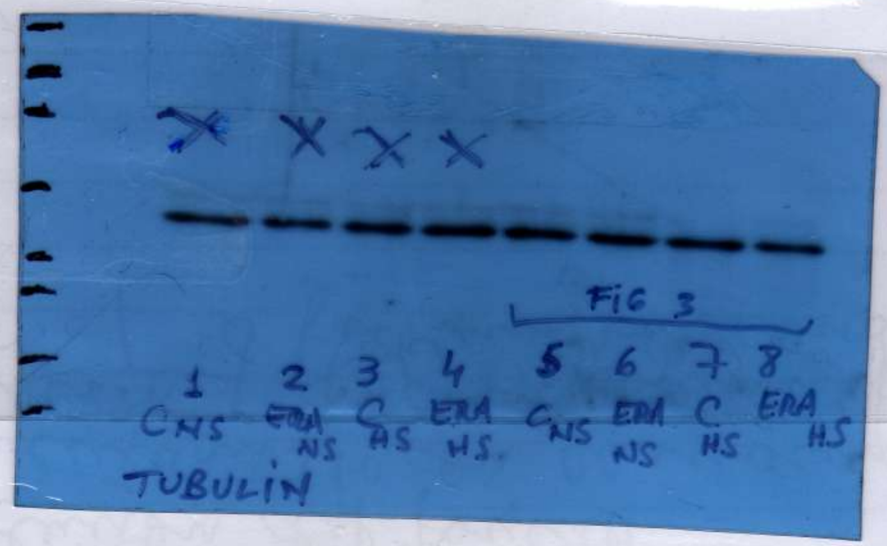

CNS = control/normosodic diet
CHS = control/ ~~norm~~ high sodium diet
ERANS = endothelin receptor antagonist treatment/normosodic d[illegible]
ERAHS = endothelin receptor antagonist treatment/high sodium [illegible]

- Method used to capture the images of the blots
epson stylus Cx 5600 Scan

- Molecular weight markers:
(tris - ClH gel)

BIO-RAD

Prestained SDS-PAGE Standards
Broad Range
Catalog 161-0318, Control 31001[illegible]

Protein	Calibrated MW (daltons) on 4-20% Tris-HCl gel	Calibrated MW (daltons) on 4-12% Bis-Tris gel
**Myosin**	**211,475**	**196,439**
**ß-Galactosidase**	**118,579**	**102,686**
**BSA**	**78,995**	**57,161**
**Ovalbumin**	**53,045**	**41,485**
**Carbonic anhydrase**	**36,881**	**27,840**
**Soybean trypsin inhibitor**	**28,643**	**20,613**
**Lysozyme**	**17,809**	**15,044**
**Aprotinin**	**6,435**	**6,445**

S 60
CL1610318 Rev D

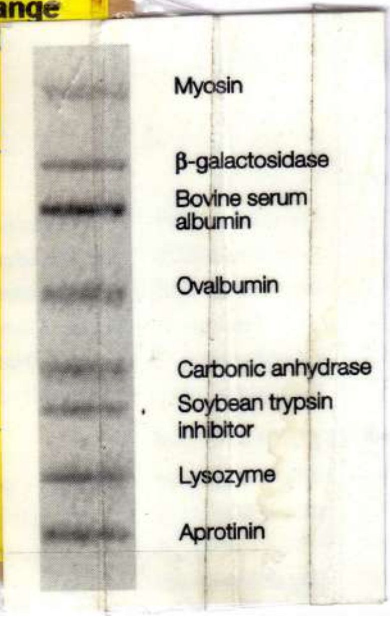

Supplement: S1 Raw images — (PDF) [file pone.0229756.s001.pdf]
