# Supplementary figures and images for: Inhibition of Endothelin system during the postnatal nephrogenic period in the rat. Its relationship with hypertension and renal disease in adulthood
Source: PLoS One. 2020 Mar 3;15(3):e0229756. doi: 10.1371/journal.pone.0229756 (PMC7053749; doi:10.1371/journal.pone.0229756)

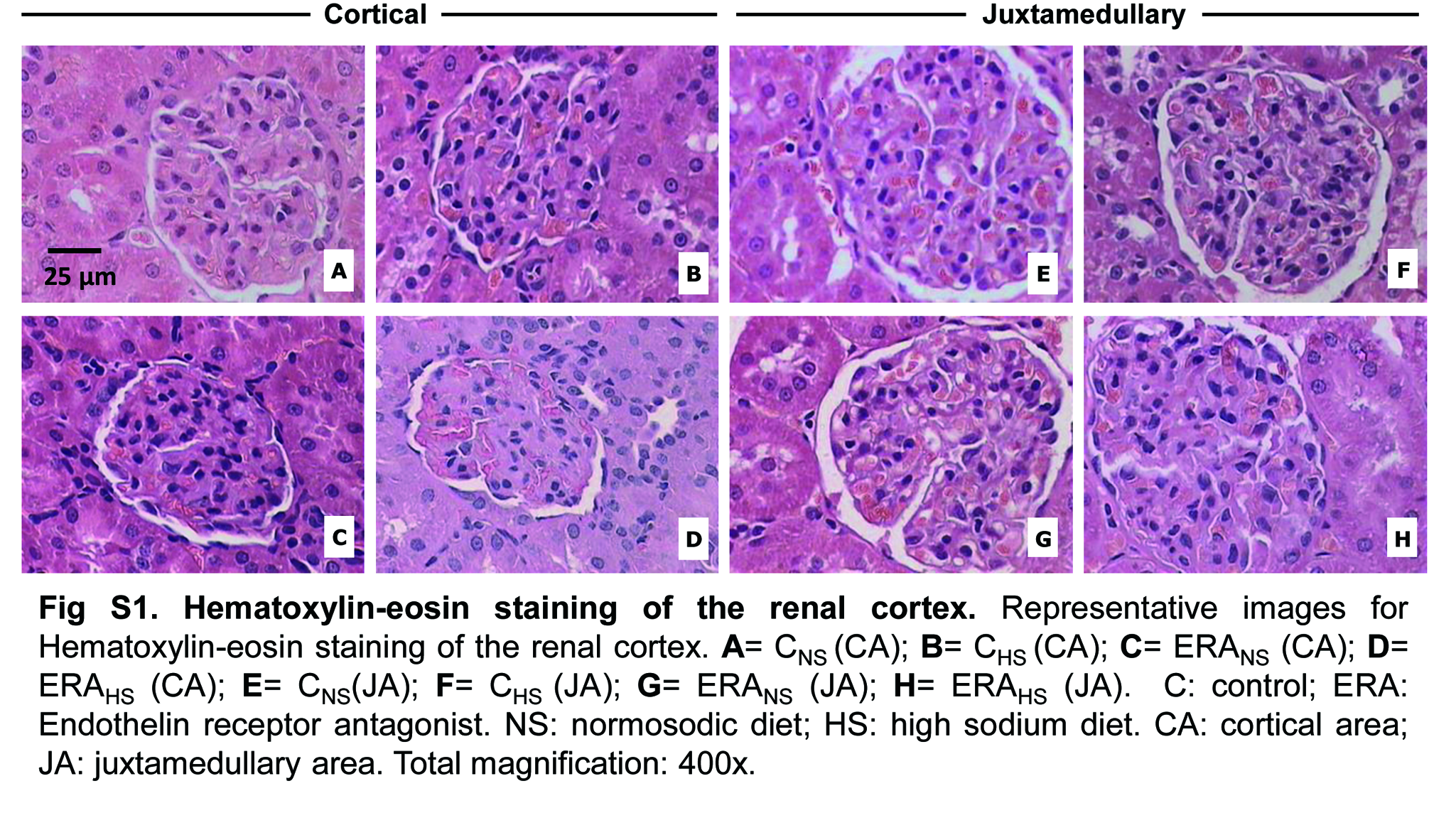

Supplement: S1 Fig — Representative images for Hematoxylin-eosin staining of the renal cortex. A = CNS (CA); B = CHS (CA); C = ERANS (CA); D = ERAHS (CA); E = CNS(JA); F = CHS (JA); G = ERANS (JA); H = ERAHS (JA). C: control; ERA: Endothelin receptor antagonist. NS: normosodic diet; HS: high sodium diet. CA: cortical area; JA: juxtamedullary area. Total magnification: 400x. (TIF) [file pone.0229756.s002.tif]

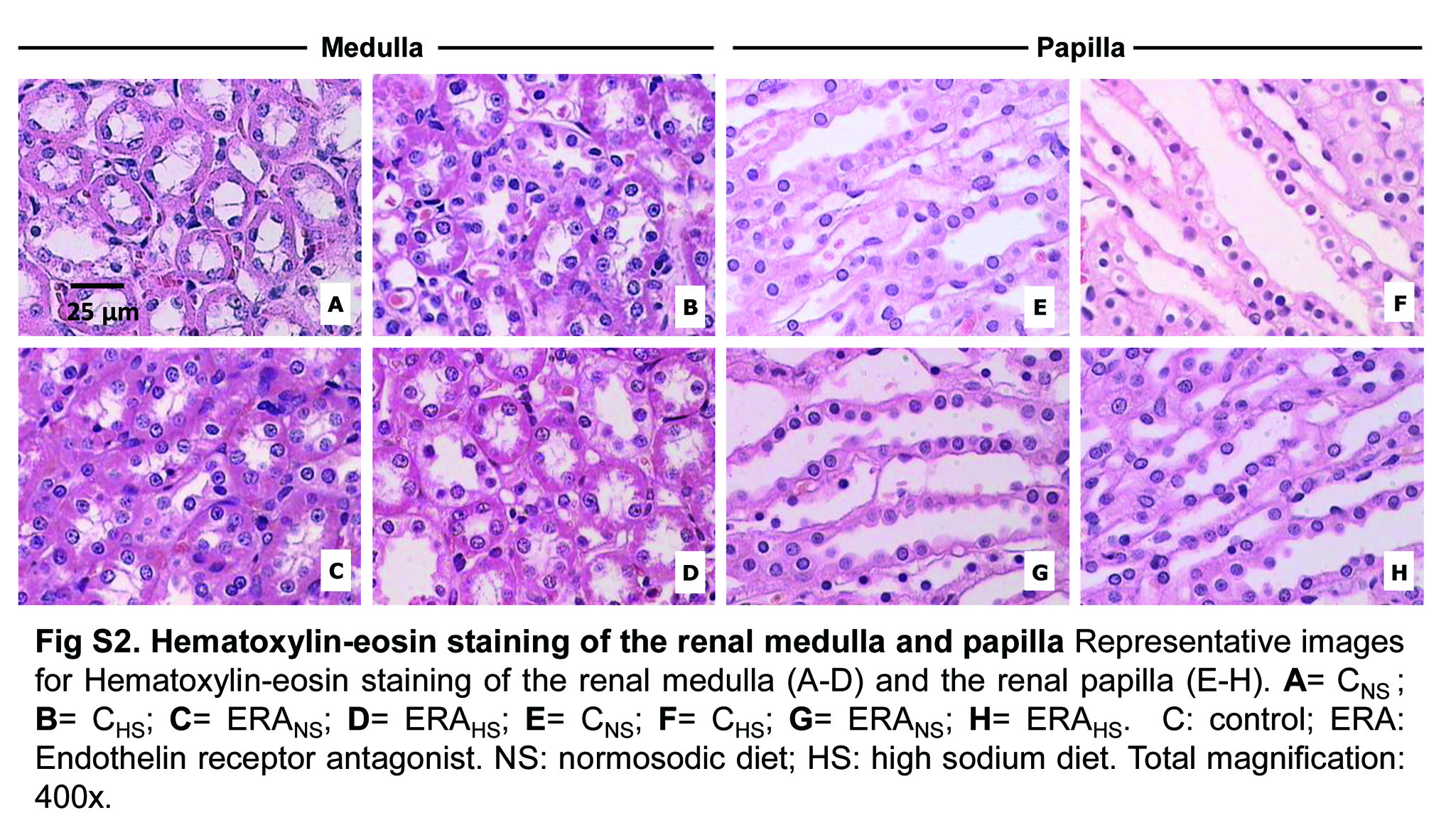

Supplement: S2 Fig — Representative images for Hematoxylin-eosin staining of the renal medulla (A-D) and the renal papilla (E-H). A = CNS; B = CHS; C = ERANS; D = ERAHS; E = CNS; F = CHS; G = ERANS; H = ERAHS. C: control; ERA: Endothelin receptor antagonist. NS: normosodic diet; HS: high sodium diet. Total magnification: 400x. (TIF) [file pone.0229756.s003.tif]

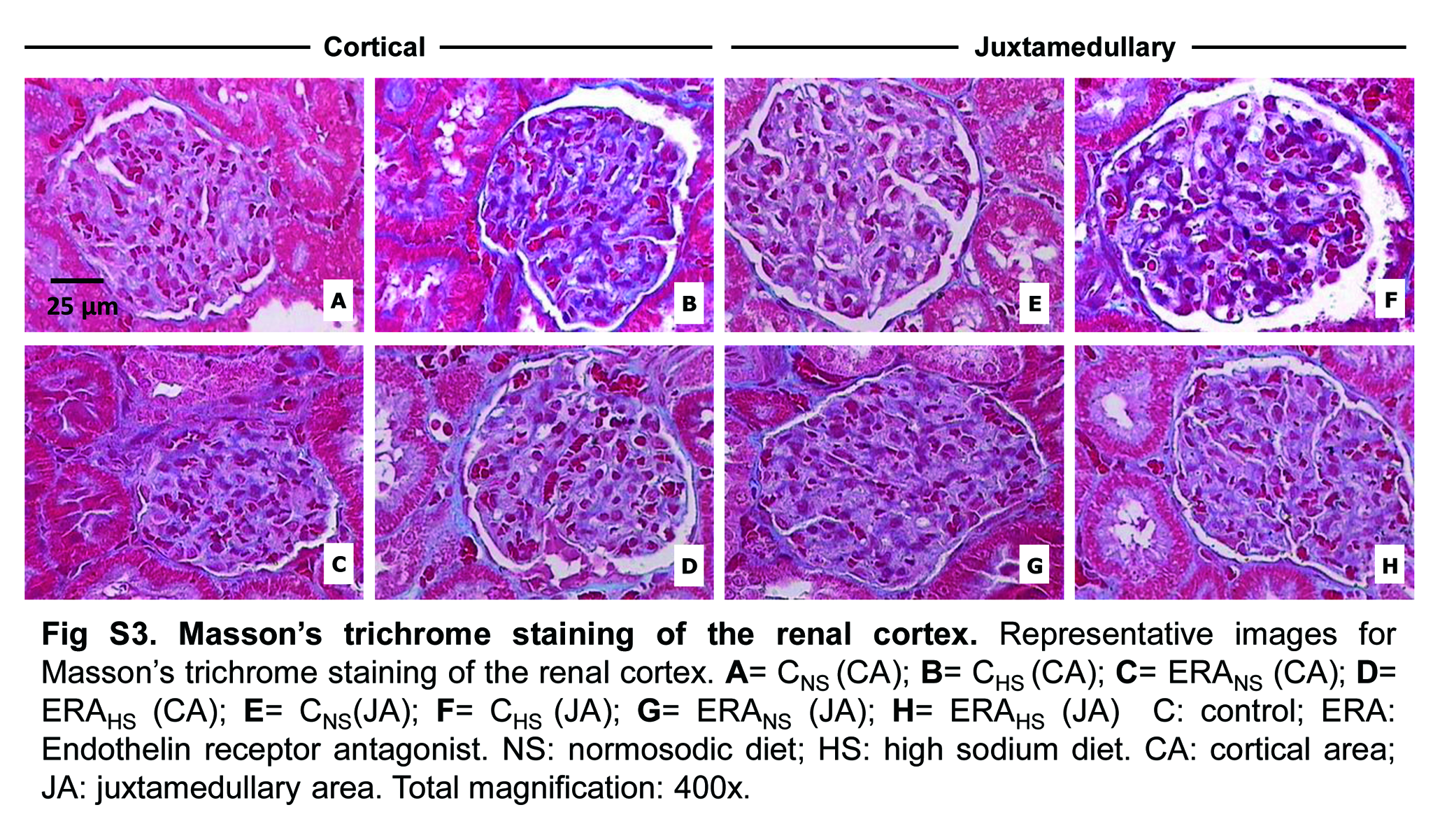

Supplement: S3 Fig — Representative images for Masson's trichrome staining of the renal cortex. A = CNS (CA); B = CHS (CA); C = ERANS (CA); D = ERAHS (CA); E = CNS(JA); F = CHS (JA); G = ERANS (JA); H = ERAHS (JA) C: control; ERA: Endothelin receptor antagonist. NS: normosodic diet; HS: high sodium diet. CA: cortical area; JA: juxtamedullary area. Total magnification: 400x. (TIF) [file pone.0229756.s004.tif]

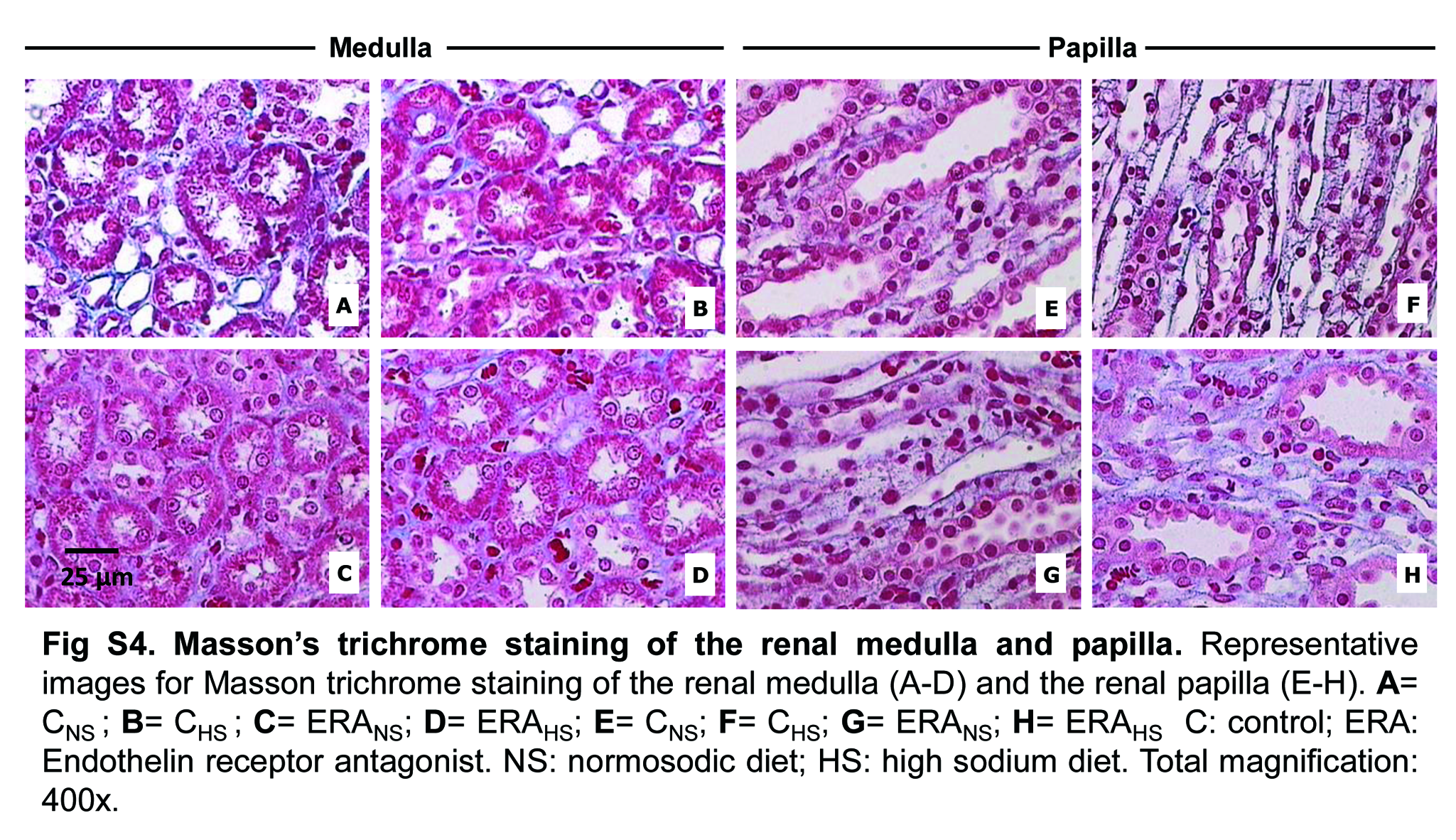

Supplement: S4 Fig — Representative images for Masson trichrome staining of the renal medulla (A-D) and the renal papilla (E-H). A = CNS; B = CHS; C = ERANS; D = ERAHS; E = CNS; F = CHS; G = ERANS; H = ERAHS C: control; ERA: Endothelin receptor antagonist. NS: normosodic diet; HS: high sodium diet. Total magnification: 400x. (TIF) [file pone.0229756.s005.tif]

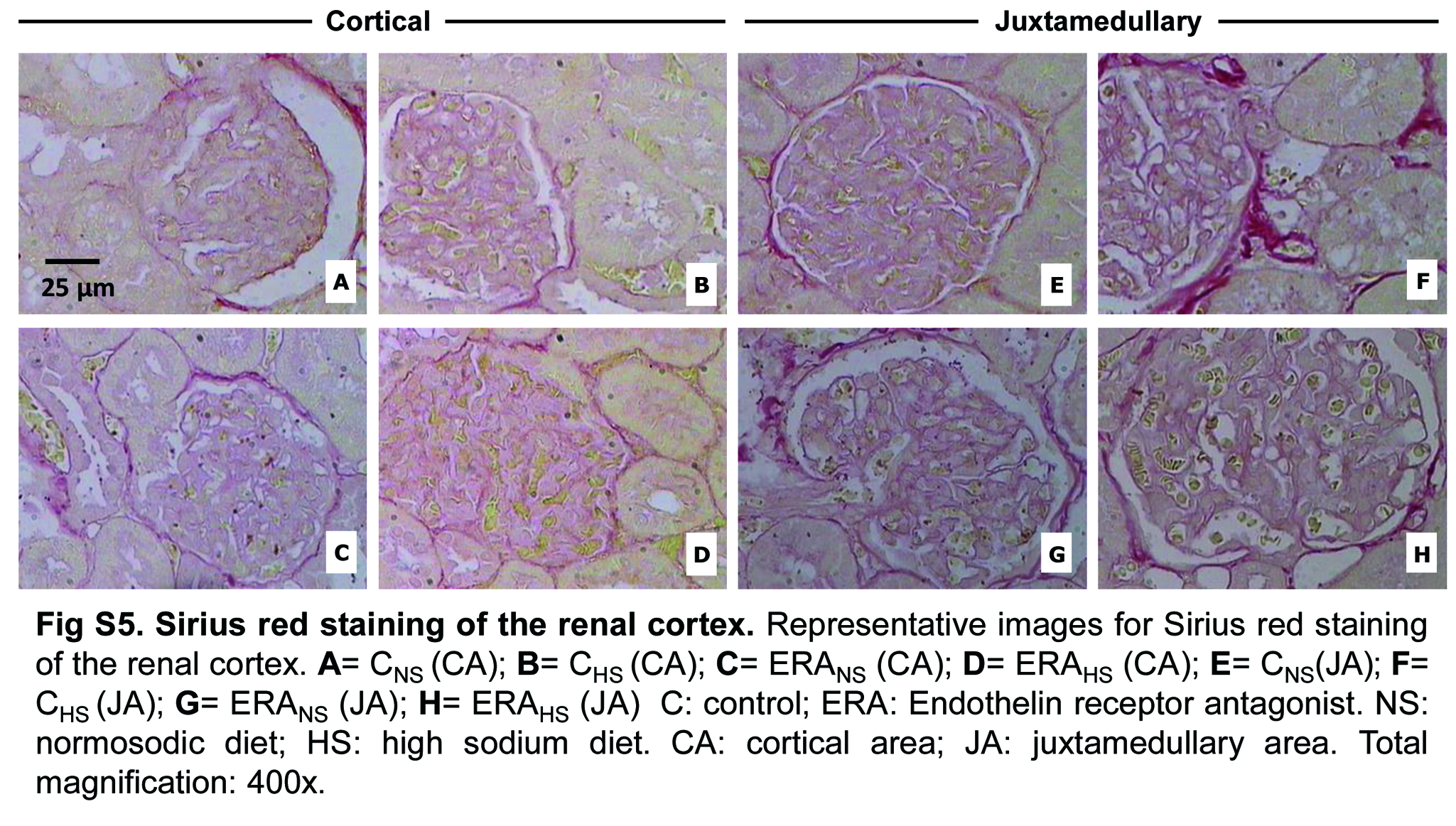

Supplement: S5 Fig — Representative images for Sirius red staining of the renal cortex. A = CNS (CA); B = CHS (CA); C = ERANS (CA); D = ERAHS (CA); E = CNS(JA); F = CHS (JA); G = ERANS (JA); H = ERAHS (JA) C: control; ERA: Endothelin receptor antagonist. NS: normosodic diet; HS: high sodium diet. CA: cortical area; JA: juxtamedullary area. Total magnification: 400x. (TIF) [file pone.0229756.s006.tif]

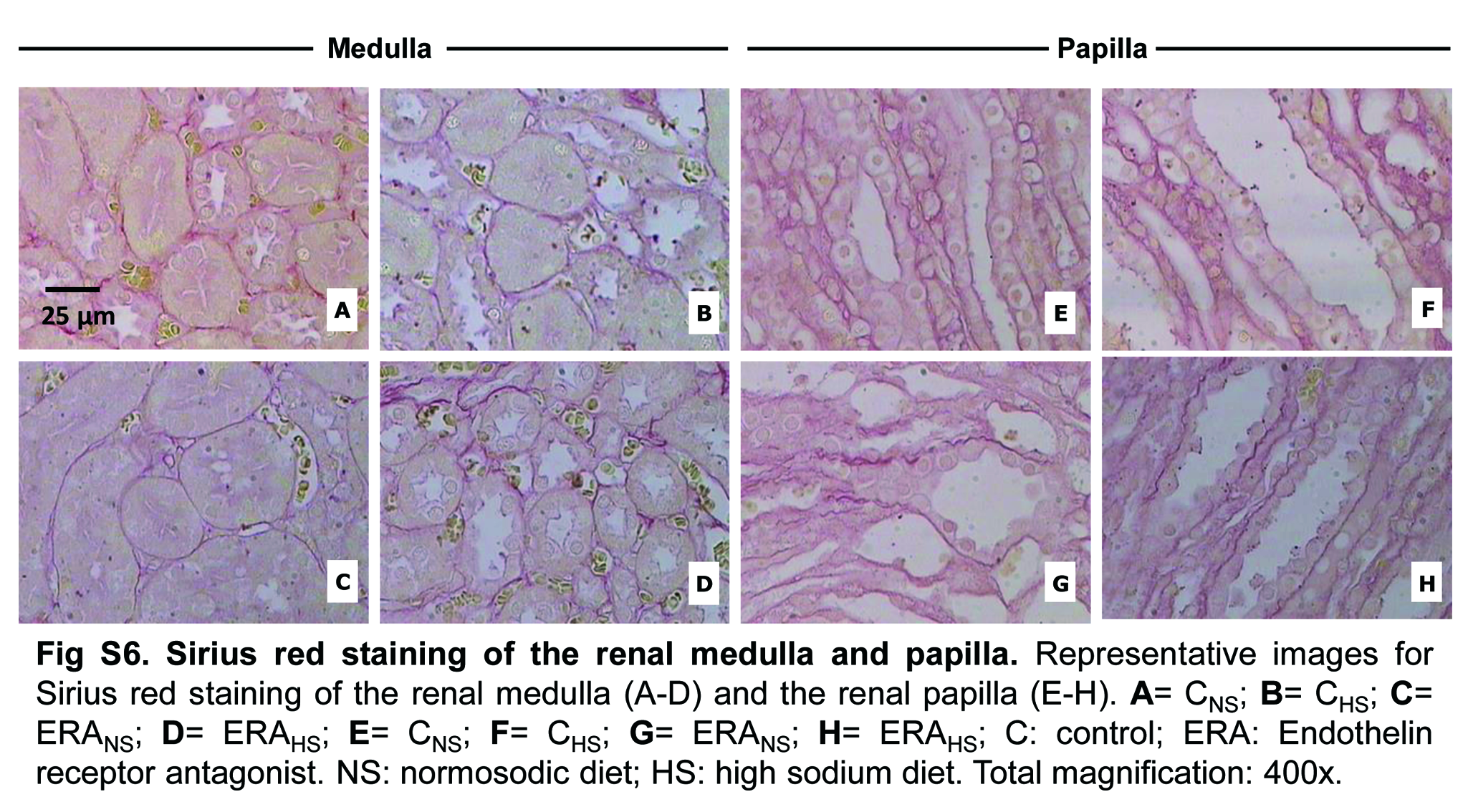

Supplement: S6 Fig — Representative images for Sirius red staining of the renal medulla (A-D) and the renal papilla (E-H). A = CNS; B = CHS; C = ERANS; D = ERAHS; E = CNS; F = CHS; G = ERANS; H = ERAHS; C: control; ERA: Endothelin receptor antagonist. NS: normosodic diet; HS: high sodium diet. Total magnification: 400x. (TIF) [file pone.0229756.s007.tif]
